# Diphyllin Shows a Broad-Spectrum Antiviral Activity against Multiple Medically Important Enveloped RNA and DNA Viruses

**DOI:** 10.3390/v14020354

**Published:** 2022-02-09

**Authors:** Michal Štefánik, Dattatry Shivajirao Bhosale, Jan Haviernik, Petra Straková, Martina Fojtíková, Lucie Dufková, Ivana Huvarová, Jiří Salát, Jan Bartáček, Jan Svoboda, Miloš Sedlák, Daniel Růžek, Andrew D. Miller, Luděk Eyer

**Affiliations:** 1 Laboratory of Emerging Viral Diseases, Veterinary Research Institute, Hudcova 296/70, CZ-621 00 Brno, Czech Republic; stefanik@vri.cz (M.Š.); bhosale@vri.cz (D.S.B.); haviernik@vri.cz (J.H.); strakova.petra@vri.cz (P.S.); fojtikova@vri.cz (M.F.); dufkova@vri.cz (L.D.); huvarova@vri.cz (I.H.); salat@vri.cz (J.S.); ruzekd@paru.cas.cz (D.R.); 2 Department of Chemistry and Biochemistry, Mendel University in Brno, Zemědělská 1665/1, CZ-613 00 Brno, Czech Republic; 3 Faculty of Chemical Technology, Institute of Organic Chemistry and Technology, University of Pardubice, Studentská 573, CZ-532 10 Pardubice, Czech Republic; jan.bartacek@upce.cz (J.B.); jan.svoboda@upce.cz (J.S.); milos.sedlak@upce.cz (M.S.); 4 Biology Centre of the Czech Academy of Sciences, Institute of Parasitology, Branišovská 1160/31, CZ-370 05 Ceske Budejovice, Czech Republic; 5 Department of Experimental Biology, Faculty of Science, Masaryk University, Kamenice 735/5, CZ-625 00 Brno, Czech Republic; 6 KP Therapeutics (Europe) s.r.o., Purkyňova 649/127, CZ-612 00 Brno, Czech Republic

**Keywords:** enveloped virus, diphyllin, cleistanthin B, vacuolar ATPase inhibitor, antiviral activity, cytotoxicity

## Abstract

Diphyllin is a natural arylnaphtalide lignan extracted from tropical plants of particular importance in traditional Chinese medicine. This compound has been described as a potent inhibitor of vacuolar (H^+^)ATPases and hence of the endosomal acidification process that is required by numerous enveloped viruses to trigger their respective viral infection cascades after entering host cells by receptor-mediated endocytosis. Accordingly, we report here a revised, updated, and improved synthesis of diphyllin, and demonstrate its antiviral activities against a panel of enveloped viruses from *Flaviviridae, Phenuiviridae, Rhabdoviridae,* and *Herpesviridae* families. Diphyllin is not cytotoxic for Vero and BHK-21 cells up to 100 µM and exerts a sub-micromolar or low-micromolar antiviral activity against tick-borne encephalitis virus, West Nile virus, Zika virus, Rift Valley fever virus, rabies virus, and herpes-simplex virus type 1. Our study shows that diphyllin is a broad-spectrum host cell-targeting antiviral agent that blocks the replication of multiple phylogenetically unrelated enveloped RNA and DNA viruses. In support of this, we also demonstrate that diphyllin is more than just a vacuolar (H^+^)ATPase inhibitor but may employ other antiviral mechanisms of action to inhibit the replication cycles of those viruses that do not enter host cells by endocytosis followed by low pH-dependent membrane fusion.

## 1. Introduction

Diphyllin **1** and diphyllin glycosides (diphyllinosides) are natural compounds of the arylnaphtalide lignan family [1,2]. These compounds were initially extracted from the plants *Cleistanthus collinus, Justicia gendarussa, Haplophyllum bucharicum,* and some others, widely used in traditional Chinese medicine [2,3,4,5]. Diphyllin **1** was originally reported to decrease potently vacuolar (H^+^)ATPase (or V-ATPase) activities in human osteoclasts [6], gastric adenocarcinoma cells [7], and in human hepatoma cells [8], plus neutralize the pH of lysosomes at nanomolar concentrations [9]. Hence, strong anticancer [10,11] and anti-inflammatory [5,12] properties have been described. Subsequently, diphyllin **1** and its derivatives have been characterized as direct inhibitors of V-ATPases [13]. 

These V-ATPases are large multisubunit protein complexes located in endosomal membranes, responsible for pumping protons into endosomal compartments while consuming ATP [14]. In cells, endosome acidification is essential for the proper functioning of the endocytic pathway, the gradual maturation of endosomes into lysosomes, vesicle trafficking, protein sorting, and the degradation of some vesicle-borne molecules [15]. Critically, endosome acidification is also known to be essential for the cell entry of some viruses, including enveloped RNA viruses, that make use of the endosome acidification process to mediate fusion of viral envelopes with endosomal membranes following the entry of virus particles into host cells via receptor-mediated endocytosis [16]. Accordingly, the inhibition of V-ATPase-mediated endosome acidification should act to inhibit these viral replication cycles. Hence, V-ATPase inhibitors could represent genuine next-generation small-molecule antiviral agents intended to abrogate viral infection processes [17]. In line with this, diphyllin **1** and diphyllinosides were found previously to exert substantial antimicrobial effects [2,18] and were found able to inhibit the replication of many fungal [19], bacterial [20], and protozoal pathogens [21]. Critically, diphyllin **1** and its glycosides were also found to inhibit the replication cycle of some enveloped RNA viruses [13,17,22,23,24,25].

Building on our previous work focused on the evaluation of the antiviral activities of diphyllin **1** against Severe Acute Respiratory Syndrome Coronavirus 2 (SARS-CoV-2) [26], we now demonstrate diphyllin **1** activity against the tick- and mosquito-borne flaviviruses, such as tick-borne encephalitis virus (TBEV), West Nile virus (WNV), and Zika virus (ZIKV). Other medically important enveloped viruses from the *Phenuiviridae* family (Rift Valley fever virus, RVFV), from the *Rhabdoviridae* family (rabies virus, RABV), and even from the *Herpesviridae* family (herpes simplex virus type 1, HSV-1), were also found susceptible to diphyllin **1** treatment. The antiviral activities and cytotoxicities of diphyllin **1** were also compared with these characteristics of a selected diphyllinoside as previously, for the sake of completeness [26]. Our results demonstrate that diphyllin **1** is a genuine, broad-spectrum antiviral agent able to abrogate infection by multiple enveloped RNA and DNA viruses. We also demonstrate that diphyllin is more than just a vacuolar (H^+^)ATPase inhibitor but may employ other antiviral mechanisms of action to inhibit the replication cycles of those viruses that do not enter host cells by endocytosis and acid pH-dependent membrane fusion. Finally, an important aspect of these studies was the creation of a revised, updated, and improved synthesis of diphyllin **1**. The primary reason for this synthesis was the cost-effective provision of diphyllin **1** in sufficient quantities for the multiple in vitro antiviral experiments described here. Other potential benefits will be outlined later. 

## 2. Materials and Methods

### 2.1. General Methods

Unless otherwise stated, reagents and solvents were purchased from commercial sources (Sigma Aldrich, St Louis, MO, USA; Acros Organics, Carlsbad, CA, USA; Merck Chemicals, Darmstadt, Germany; TCI Europe, Zwijndrecht, Belgium). Commercial grade reagents were used without further purification. Reactions were monitored by thin-layer chromatography plates coated with 0.2 mm silica gel 60 F254 (Merck Chemicals, Darmstadt, Germany). TLC plates were visualized by UV irradiation (254 nm). All melting points were determined on a Melting Point B-540 apparatus (Büchi, Flawil, Switzerland) and are uncorrected. IR spectra were recorded on a Nicolet 6700FT-IR spectrometer (Thermo Fisher Scientific, Waltham, MA, USA) over the range of 400–4000 cm^−1^ using the ATR technique. NMR spectra were measured in CDCl_3_, DMSO-d_6_ solution at ambient temperature on a Bruker Avance^TM^ III 400 spectrometer at frequencies ^1^H (400 MHz) and ^13^C (100.26 MHz) or a Bruker Ascend^TM^ 500 spectrometer at frequencies ^1^H (500.13 MHz), ^13^C (125.76 MHz) (Bruker, Billerica, MA, USA). The chemical shift, δ, was measured relative to CDCl_3_ 7.27 p.p.m. or DMSO-d_6_ 2.5 p.p.m. using tetramethylsilane (TMS) as an internal standard. Coupling constants (*J*) were all reported in (Hz). Elemental analysis (C, H, N) was performed on an automatic microanalysis Flash 2000 Organic elemental analyzer (Thermo Fisher Scientific, Waltham, MA, USA). Mass spectrometry with high resolution was determined using the “dried droplet” method with a MALDI mass spectrometer LTQ Orbitrap XL (Thermo Fisher Scientific, Waltham, MA, USA) equipped with a nitrogen laser (337 nm, 60 Hz). Spectra were measured in the positive ion mode and in regular mass extent at a resolution of 100,000 at *m/z* 400. The matrix used was 2,5-dihydrobenzoic acid (DBH).

### 2.2. Synthesis of benzo[d][1,3]dioxole-5-carbaldehyde ***2***

To a solution of 3,4-dihyroxybenzaldehyde (13.8 g, 99 mmol) in cooled acetonitrile (CH_3_CN) (200 mL), were added potassium carbonate (K_2_CO_3_) (40 g, 289 mmol) and dibromomethane (10,4 mL, 119 mmol). The reaction mixture was then stirred at 90 °C for 24 h. After completion (monitored by TLC), the reaction mixture was cooled to room temperature and filtered. The filtrate was concentrated then extracted with ethyl acetate and water. Thereafter, the organic layer was dried over anhydrous sodium sulfate, concentrated, and the residue purified by column chromatography eluting with ethyl acetate:*n*-hexane (1:1, *v/v*) to yield compound **2** (14 g, 94%) as a brown solid. Mp 40–42 °C. IR(ATR): 3081, 3063, 2998, 2985, 2918, 2851, 2793, 2752, 2722, 1669, 1599, 1487, 1446, 1253, 1035, 927, 864, and 811 cm^−1^. ^1^H NMR (CDCl_3_, 500.13 MHz): δ 9.80 (1H, s, -C*H*O), 7.41 (1H, dd, *J* = 1.5 Hz, *J* = 8.0 Hz, Ar-*H*), 7.33 (1H, d, *J* = 1.5 Hz, Ar-*H*), 6.99 (1H, d, *J =* 8.0 Hz, Ar-*H*), and 6.07 (2H, s, -O-C*H_2_*-O-). ^13^C NMR (CDCl_3_, 125.67 MHz): δ 190.4, 153.2, 148.8, 131.9, 128.8, 108.4, 107.0, and 102.2. CHN analysis: Calculated for C_8_H_6_O_3_ (150.13): C, 64.00; H, 4.03. Found: C, 64.07 ± 0.01; H, 4.10 ± 0.02. HRMS: *m/z* calculated for C_8_H_6_O_3_: 151.03897 [M+H]^+^; found: 151.03925 [M+H]^+^.

### 2.3. Synthesis of 2-(2-bromo-4,5-dimethoxyphenyl-[1,3]dithiane ***3***

To a round bottom flask containing dry benzene (300 mL) were added 2-bromo-4,5-dimethoxybenzaldehyde (12.3 g, 50.2 mmol), 1,3-propanedithiol (5.04 mL, 50.2 mmol), and *p*-toluenesulfonic acid (*p*-TsOH) (0.48 g, 2.79 mmol). The flask was then connected to a Dean-Stark trap and the reaction mixture heated under reflux for 10 h. After completion (monitored by TLC), the reaction mixture was cooled to room temperature, solvent was removed under reduced pressure, and the residue was partitioned between diethyl ether (100 mL), 1M NaOH (100 mL), water (200 mL), and brine (200 mL). The organic layer was subsequently dried over anhydrous sodium sulfate, concentrated, and the residue was purified by column chromatography eluting with ethyl acetate:*n*-hexane (1:3, *v/v*) to yield compound **3** (16.7 g, 100%) as a white solid. Mp 125–126 °C. IR (ATR): 3068, 2996, 2965, 2899, 1598, 1505, 1484, 1461, 1449, 1379, 1259, 1250, 1 028, 968, 906, 906, 808, 779, 760, and 578 cm^−1^. ^1^H NMR (CDCl_3_, 400 MHz): δ. 7.15 (1H, s, Ar-*H*), 6.97 (1H, s, Ar-*H*), 5.52 (1H, s, -CH_2_-S-C*H-*Ar), 3.89 (3H, s, -OC*H_3_*), 3,84 (3H, s, -OC*H_3_*), 3.12 (2H, brd, *J* = 12.4 Hz, -S-C*H_2_*-CH_2_-CH_2_-S-), 2,90 (2H, brdt *J* = 14.8, 2.8 Hz, -S-CH_2_-CH_2_-C*H_2_*-S-), 2.17 (1H, m, -S-CH_2_-C*H*H-CH_2_-S-), and 1.93 (1H, m, -S-CH_2_-CH*H*-CH_2_-S-). ^13^C NMR (CDCl_3_, 100.26 MHz): δ 149.4, 149.0, 130.2, 115.3, 113.0, 111.1, 56.29, 56.28, 49.3, 32.4, and 25.1. CHN analysis: Calculated for C_12_H_15_BrO_2_S_2_ (335.28): C, 42.99; H, 4.51; S, 19.13. Found: C, 43.37 ± 0.28; H, 4.45 ± 0.02; S, 18.63 ± 0.14. HRMS: *m/z* calculated for C_12_H_15_BrO_2_S_2_: 334.97696 [M+H]^+^; found: 334.97817 [M+H]^+^.

### 2.4. Synthesis of benzo[1,3]dioxol-5-yl-(2-[1,3]dithian-2-yl-4,5-dimethoxyphenyl)-methanol ***4***

Dithiane **3** (14 g, 41.7 mmol) was dissolved in dry tetrahydrofuran (THF) (200 mL), then *n*-butyl lithium (*n*-BuLi) (39 mL, 1.6 mL solution in *n*-hexane, 62.6 mmol) was added at −78 °C under a N_2_ atmosphere, after which the reaction mixture was stirred for 1 h. A solution of piperonal **2** (6.89 g, 45.9 mmol) in dry THF (30 mL) was further added at −78 °C, then the reaction mixture was stirred for 2 h, before warming to room temperature over 3 h. After completion (monitored by TLC), the reaction mixture was quenched with a saturated solution of ammonium chloride (90 mL), then extracted with ethyl acetate and water. Thereafter the organic layer was further extracted with brine solution (150 mL), dried over anhydrous sodium sulfate, and evaporated under reduced pressure, giving a crude product residue that was purified by flash chromatography eluting with ethyl acetate:*n*-hexane (1:3, *v/v*) to yield compound **4** (15 g, 89%) as a white solid. Mp 75–76 °C. IR (ATR): 3491, 3076, 2996, 2933, 2894, 2848, 2776, 1606, 1509, 1486, 1446, 1440, 1235, 1167, 1082, 1034, 925, 894, 821, 755, 667 cm^−1^. ^1^H NMR (CDCl_3_, 400 MHz): δ. 7.12 (1H, s, Ar-*H*), 6.88 (1H, d, *J* = 1.6 Hz, Ar-*H*), 6.85 (1H, brd, *J* = 8.0, 1.2 Hz, Ar-*H*), 6.82 (1H, s, Ar-*H*), 6.78 (1H, d, *J* = 8 Hz, Ar-*H*), 6.14 (1H, s, -Ar-C*H*-OH-Ar), 5.94 (2H, s, -O-C*H_2_*-O-), 5.39 (1H, s, -CH_2_-S-C*H-*Ar), 3.90 (3H, s, -OC*H_3_*), 3.79 (3H, s, -OC*H_3_*), 2.97 (2H, brtd, *J* = 9.6, 6 Hz, -S-C*H_2_*-CH_2_-CH_2_-S-), 2.85 (2H, brdt, *J* = 14.4, 3.6 Hz, -S-CH_2_-CH_2_-C*H_2_*-S-), 2.58 (1H, s, -Ar-CH-O*H*-Ar), 2.13 (1H, m, -S-CH_2_-C*H*H-CH_2_-S-), 1.89 (1H, m, -S-CH_2_-CH*H*-CH_2_-S-). ^13^C NMR (CDCl_3_, 100.26 MHz): δ 148.96, 148.93, 147.8, 146.9, 137.2, 133.4, 129.0, 120.0, 111.5, 110.6, 108.1, 107.4, 101.1, 72.1, 56.6, 56.0, 48.0, 32.67, 32.60, 25.1. CHN analysis: Calculated for C_20_H_22_O_5_S_2_ (406.52): C, 59.09; H, 5.45; S, 15.78. Found: C, 58.79 ± 0.15; H, 5.32 ± 0.01; S, 15.63 ± 0.08. HRMS: *m/z* calculated for C_20_H_22_O_5_S_2_: 407.09814 [M+H]^+^; 429.08009 [M+Na]^+^; found: 407.09865 [M+H]^+^; 429.08237 [M+Na]^+^.

### 2.5. Synthesis of benzo[1,3]dioxol-5-yl-(2-[1,3]dithian-2-yl-4,5-dimethoxyphenyl)-methanone ***5***

Dimethoxyphenyl alcohol **4** (13 g, 32 mmol) was dissolved in dichloromethane (CH_2_Cl_2_) (200 mL), then activated manganese dioxide (MnO_2_) (48 g, 552 mmol) added. Thereafter the reaction mixture was stirred at room temperature under a N_2_ atmosphere for 16 h. After completion (monitored by TLC), the reaction mixture was filtered through a plug of Celite and washed with dichloromethane (400 mL). Thereafter, the filtrate was evaporated under reduced pressure to dryness giving compound **5** (12 g, 93%) as a white solid. Mp 150–152 °C. IR (ATR): 3065, 3004, 2918, 2901, 2848, 2828, 1656, 1600, 1513, 1483, 1464, 1436, 1237, 1178, 1033, 955, 892, 827, 765, and 582 cm^−1^. ^1^H NMR (CDCl_3_, 400 MHz): δ 7.35 (1H, d, *J* = 0.4 Hz, Ar-*H*), 7.31 (1H, dd, *J* = 8.0, 2.0 Hz, Ar-*H*), 7,26 (1H, s, Ar-*H*), 6.79 (1H, d, *J* = 8.0 Hz, Ar-*H*), 6.75 (1H, s, Ar-*H*), 6.02 (2H, s, -O-C*H_2_*-O-), 5.39 (1H, s, -CH_2_-S-C*H-*Ar), 3.94 (3H, s, -OC*H_3_*), 3.77 (3H, s, -OC*H_3_*), 2.89 (2H, brtd, *J* = 12.4, 2.4 Hz, -S-C*H_2_*-CH_2_-CH_2_-S-), 2.77 (2H, brdt, *J* = 14.4, 3.6 Hz, -S-CH_2_-CH_2_-C*H_2_*-S-), 2.05 (1H, m, -S-CH_2_-C*H*H-CH_2_-S-), and 1.84 (1H, m, -S-CH_2_-CH*H*-CH_2_-S-). ^13^C NMR (CDCl_3_, 100.26 MHz): δ 195.0, 152.1, 151.1, 148.1, 147.1, 132.7, 131.6, 129.8, 127.6, 112.1, 111.9, 109.6, 107.8, 102.0, 56.29, 56.22, 47.6, 32.3, and 25.1. CHN analysis: Calculated for C_20_H_20_O_5_S_2_ (404.50): C, 59.39; H, 4.98; S, 15.85. Found: C, 60.01 ± 0.10; H, 4.99 ± 0.02; S, 15.67 ± 0.05. HRMS: *m/z* calculated for C_20_H_20_O_5_S_2_: 405.08249 [M+H]^+^; 427.06444 [M+Na]^+^; found: 405.08321 [M+H]^+^; 427.06552 [M+Na]^+^. 

### 2.6. Synthesis of 9-benzo[1,3]dioxol-5-yl-9-hydroxy-4-([1,3]dithian-2-yl)-6,7-dimethoxy-3a,4, 4,9a-tetrahydro-3H-naphtho[2,3-c]furan-1-one ***6***

Benzophenone intermediate **5** (12 g, 29.7 mmol) was dissolved in dry THF (200 mL), lithium hexamethyldisilazide (LiHMDS) (1.0 M in THF, 39 mL) added at −65 °C under a N_2_ atmosphere, and then the reaction mixture was stirred at −65 °C for 4 h to generate a deep purple anion. Following this, a solution of 2(5H)-furanone (3.2 g, 38.6 mmol, in dry THF) was then added drop by drop over 15 min. Thereafter, the reaction mixture was stirred at −65 °C under a N_2_ atmosphere for another 48 h. After completion (monitored by TLC), the reaction mixture was warmed to room temperature for 1 h and quenched with H_2_O (5 mL), before the organic solvent was evaporated under reduced pressure. The resulting crude product residue was then purified by column chromatography eluting with ethyl acetate:*n*-hexane (4:1, *v/v*) to yield compound **6** (3.7 g, 26%) as a white solid. Mp 205–207 °C. IR (ATR): 3503, 3087, 2973, 2935, 2904, 2847, 2825, 1766, 1741, 1608, 1514, 1502, 1481, 1464, 1454, 1435, 1372, 1361, 1301, 1263, 1235, 1173, 1081, 1062, 1018, 973, 948, 893, 818, 785, 729, and 636 cm^−1^. ^1^H NMR (DMSO-d_6_, 500.13 MHz): δ 7.43 (1H, s, Ar-*H*), 6.86 (1H, d, *J* = 8.0 Hz, Ar-*H*), 6.80 (1H, d, *J* = 1.5 Hz, Ar-*H*), 6.74 (1H, dd, *J* = 7.0, 2.0 Hz, Ar-*H*), 6.40 (1H, s, Ar-*H*), 6.08 (1H, s, -Ar-C-O*H*-CH-), 6.00 (2H, s, -O-C*H_2_*-O-), 4.30 (1H, m, -C-C*H*-CH_2_-O-), 4.06 (2H, m, -C-CH-C*H_2_*-O-), 3.77 (3H, s, -OC*H_3_*), 3.51 (3H, s, -OC*H_3_*), 3.43 (1H, brtd, *J* = 13.7, 2.5 Hz, -S-CH_2_-CH_2_-CH*H*-S-), 3.27 (1H, brt, *J* = 13.5 Hz, -S-CH_2_-CH_2_-C*H*H-S-), 3.08 (1H, brd, *J* = 6.0 Hz, -Ar-C-OH-C*H*-CO), 2.87 (1H, brd, *J* = 15.0 Hz, -S-C*H*H-CH_2_-CH_2_-S-), 2.71 (1H, brd, *J* = 14.5 Hz, -S-CH*H*-CH_2_-CH_2_-S-), 2.17 (1H, brd, *J* = 14.5 Hz, -S-CH_2_-CH*H*-CH_2_-S-), and 1.77 (1H, brq, *J* = 13.0 Hz, -S-CH_2_-C*H*H-CH_2_-S-). ^13^C NMR (DMSO-d_6_, 125.76 MHz): δ 174.2, 148.7, 148.5, 146.7, 145.7, 143.6, 133.8, 126.4, 119.3, 112.5, 109.4, 107.5, 106.7, 101.0, 69.9, 68.3, 55.5, 55.3, 51.0, 49.0, 41.0, 28.1, 26.2, and 23.7. CHN analysis: Calculated for C_24_H_24_O_7_S_2_ (488.57): C, 59.00; H, 4.95; S, 13.13. Found: C, 58.68 ± 0.18; H, 5.23 ± 0.02; S, 12.82 ± 0.07. HRMS: *m/z* calculated for C_24_H_24_O_7_S_2_: 511.08557 [M+Na]^+^; 527.05950 [M+K]^+^; found: 511.08816 [M+Na]^+^; 527.06223 [M+K]^+^.

### 2.7. Synthesis of 9-benzo[1,3]dioxol-5-yl-9-hydroxy-6,7-dimethoxy-3,3a,9,9a-tetrahydronaph- -tho[2,3-c]furan-1,4-dione ***7***

A solution of lactone **6** (3.5 g, 7.1 mmol) in aqueous acetonitrile (85%, *v/v*, 50 mL) with mercuric oxide (HgO) (1.6 g, 7.8 mmol) and mercury chloride (HgCl_2_) (4.2 g, 15.7 mmol) was heated under reflux for 5 h. After completion (monitored by TLC), the reaction mixture was cooled to room temperature, filtered through a Celite bed, and the filtrate evaporated under reduced pressure. The resulting crude product residue was then dissolved in chloroform (150 mL), and extracted with a saturated solution of ammonium carbonate. Thereafter the organic layer was extracted with brine solution (150 mL), dried over anhydrous sodium sulfate and evaporated under reduce pressure. The resulting crude product residue was purified by flash chromatography eluting with ethyl acetate:*n*-hexane (2:3, *v/v*) to yield compound **7** (1.257 g, 44.2%) as a white solid. Mp 103–104 °C. IR (ATR): 3451, 2966, 2919, 2850, 2640, 1747, 1735, 1665, 1593, 1505, 1484, 1460, 1436, 1364, 1320, 1278, 1239, 1212, 1145, 1129, 1096, 1080, 1031, 1010, 987, 904, 820, 794, 765, 723 cm^−1^. ^1^H NMR (CDCl_3_, 400 MHz): δ 7.48 (1H, s, Ar-*H*), 7.25 (1H, s, Ar-*H*), 6.86 (1H, d, *J* = 2.0 Hz, Ar-*H*), 6.65 (1H, d, *J* = 8.0 Hz, Ar-*H*), 6.41 (1H, dd, *J* = 8.4, 2.5 Hz, Ar-*H*), 5.94 (2H, s, -O-C*H_2_*-O-), 5.74 (1H, s, -Ar-C-O*H*-CH-), 4.73 (1H, d, *J* = 9.6 Hz,-CO-CH-C*H*H-O-), 4.30 (1H, dd, *J* = 9.2, 5.6 Hz, -CO-CH-CH*H*-O-), 3.96 (3H, s, -OC*H*_3_), 3.94 (3H, s, -OC*H*_3_), 3.44 (1H, d, *J* = 7.2 Hz, -Ar-C(OH)-C*H*-CO-O-), and 3.09 (1H, dd, *J* = 5.6, 4.8 Hz,-CO-C*H*-CH_2_-O-). ^13^C NMR (CDCl_3_, 100.26 MHz): δ 193.0, 176.9, 155.9, 149.7, 148.2, 147.6, 141.2, 138.5, 125.5, 120.1, 108.8, 108.1, 107.9, 107.0, 101.5, 72.5, 70.8, 56.5, 56.2, 50.4, and 46.1. CHN analysis: Calculated for C_21_H_18_O_8_ (398.36): C, 63.32; H, 4.55. Found: C, 63.21 ± 0.03; H, 4.69 ± 0.01. HRMS: *m/z* calculated for C_21_H_18_O_8_: 421.08939 [M+Na]^+^; 437.06333 [M+K]^+^; found: 421.09018 [M+Na]^+^; 437.06448 [M+K]^+^. 

### 2.8. Synthesis of 9-(3‣,4‣-methylenedioxyphenyl)-4-hydroxy-6,7-dimethoxynaphtho[2,3-c]furan-1(3H)-one (Diphyllin) ***1***

Benzofuranone **7** (1.084 g, 2.7 mmol) and *p*-TsOH (0.362 g, 1.9 mmol) were heated under reflux in benzene (80 mL) for 15 h. After completion (monitored by TLC), the reaction mixture was cooled to room temperature and the solvent removed under reduced pressure. The crude product residue was then purified by flash chromatography eluting with ethyl acetate:*n*-hexane (1:1, *v/v*) to yield compound **1** (0.730 g, 70%) as a yellow solid that was recrystallized. Mp 275–277 °C. IR (ATR): 3197, 3006, 2955, 2923, 2853, 2648, 1703, 1613, 1594, 1507, 1492, 1455, 1433, 1358, 1332, 1227, 1211, 1192, 1169, 1125, 1084, 1035, 1007, 930, 948, 915, 860, 768, 730, and 719 cm^−1^. ^1^H NMR (DMSO-d_6_, 400 MHz): δ. 10.4 (1H, s, Ar-O*H*), 7.61 (1H, s, Ar-*H*), 7.01 (1H, d, *J* = 8.0 Hz, Ar-*H*), 6.95 (1H, s, Ar-*H*), 6.86 (1H, d, *J* = 1.6 Hz, Ar-*H*), 6.75 (1H, dd, *J* = 10.0, 5.6 Hz, Ar-*H*), 6.11 (2H, s, -O-C*H_2_*-O), 5.35 (2H, s, -O-C*H_2_*-Ar), 3.94 (3H. s, -OC*H*_3_), 3.65 (3H, s, -OC*H*_3_). ^13^C NMR (DMSO-d_6_, 100.26 MHz): δ 169.6, 150.4, 149.6, 146.8, 146.6, 144.8, 129.5, 129.4, 128.8, 123.7, 123.2, 121.6, 118.6, 111.0, 107.8, 105.4, 101.0, 100.7, 66.6, 55.5, and 55.1. CHN analysis: Calculated for C_21_H_16_O_7_ (380.35): C, 66.31; H, 4.24. Found: C, 66.45 ± 0.10; H, 4.56 ± 0.02. HRMS: *m/z* calculated for C_21_H_16_O_7_: 381.09688 [M+H]^+^; 403.07882 [M+Na]^+^; 419.05276 [M+K]^+^; found: 381.09809 [M+H]^+^; 403.08028 [M+Na]^+^; 419.05421 [M+K]^+^. Previously reported data [M−H]^+^ 379.0825 [24].

### 2.9. Viruses, Cells, and Compounds

Our antiviral studies were performed using typical representatives of the following virus families: (i) *Flaviviridae* (TBEV, strain Hypr, a representative of the European TBEV subtype, provided by the Collection of Arboviruses, Institute of Parasitology, Biology Centre of the Czech Academy of Sciences, Ceské Budějovice, Czech Republic (http://www.arboviruscollection.cz/index.php?lang=en, accessed on 10 January 2022); ZIKV, the Brasilian strain Paraiba_01; kindly provided by Prof. Paolo M. de A. Zanotto, University of São Paulo, Brasil; WNV, strain Eg-101, provided by the Collection of Arboviruses, Institute of Parasitology, Biology Centre of the Czech Academy of Sciences, Ceske Budejovice, Czech Republic); (ii) *Phenuiviridae* (RFVF, strain H13/96, kindly provided by Karel Bilek, National Institute of Nuclear, Chemical and Biological Protection, Czech Republic); (iii) *Rhabdoviridae* (RABV, strain CVS-11, kindly provided by Prof. Anthony R. Fooks, Animal and Plant Health Agency, UK); and (iv) *Herpesviridae* (HSV-1, strain MacIntyre, kindly provided by Prof. Andreas Sauerbrei, German Reference Laboratory of HSV und VZV, Germany). 

Vero cells (ATCC CCL-81, African Green Monkey, adult kidney, epithelial), baby hamster kidney cells (BHK-21, ATTC CCL-10), and human hepatocarcinoma cells (Huh-7) were grown in Dulbecco′s modified Eagle′s medium (DMEM); porcine kidney stable (PS) [27] were cultured in Leibovitz (L-15) medium; human brain cortical astrocytes (HBCA, ScienCell, Carlsbad, CA, USA) were cultivated in Astrocyte medium; human neuroblastoma UKF-NB-4 cells [28] were cultured in Iscove’s modified Dulbecco’s medium (IMDM). The media were supplemented with 3% (L-15), 6% (Astrocyte medium), or 10% (IMDM and DMEM) newborn calf serum plus 100 U/mL penicillin, 100 µg/mL streptomycin, and 1% glutamine (Sigma-Aldrich, Prague, Czech Republic). Vero, HBCA, UKV-NB-4, Huh-7, and BHK-21 cells were cultured at 37 °C under 5% CO_2_, whereas PS cells were cultivated at 37 °C under a normal atmosphere (without CO_2_ supplementation). 

**Scheme 1:**
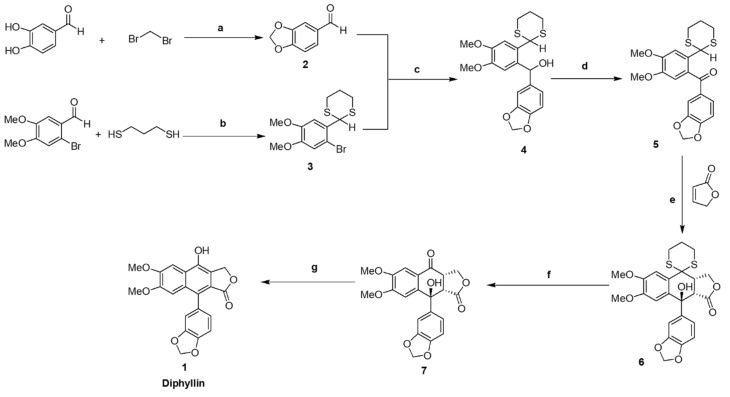
Synthesis of diphyllin **1**. Reagents and conditions: (**a**) K_2_CO_3_, CH_3_CN, N_2_, 90 °C, 24 h, 94%; (**b**) *p*-TsOH, dry-benzene, reflux, 10 h, 100%; (**c**) *n*-BuLi, dry-THF, N_2_, −78 °C for 2 h and rt, 3 h, 89%; (**d**) MnO_2_, CH_2_Cl_2_, N_2_, rt, 16 h, 93%; (**e**) LiHMDS, dry-THF, N_2_, −65 °C, 48 h, 26%; (**f**) HgO, HgCl_2_, dry-CH_3_CN, reflux, 5 h, 44%; (**g**) *p*-TsOH, benzene, reflux, 15 h, 70%.

**Figure 1:**
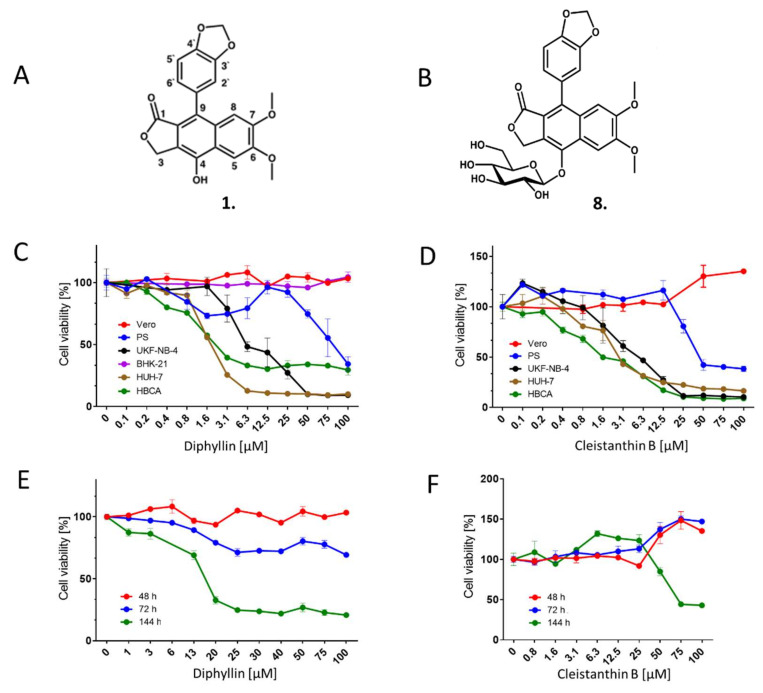
Cytotoxicity of diphyllin **1** and cleistanthin B **8**. (**A**) Structure of diphyllin **1**; atom numbering is included. (**B**) Structure of cleistanthin B **8**. (**C**) Cytotoxicities of diphyllin **1** for Vero, PS, UKF-NB-4, BHK-21, Huh-7, and HBCA were expressed as a percentage of cell viability at the indicated drug concentration. The cells were seeded in 96-well plates for 24 h, then treated with diphyllin **1** and incubated for 48 h. Diphyllin **1**-treated BHK-21 cells were incubated for 72 h. Cell viabilities were measured by Cell Counting Kit-8. (**D**) Cytotoxicities of cleistanthin B **8** were expressed as percentage cell viability at the indicated drug concentration. The experimental procedure was the same as in (**C**). (**E**) Time-dependent cytotoxic effects of diphyllin **1** on Vero cells. Vero cells were seeded in 96-well plates for 24 h, then treated with diphyllin **1** at the indicated concentrations. Cell viabilities were determined after 48, 72, and 144 h of incubation. (**F**) Time-dependent cytotoxic effects of cleistanthin B **8** on Vero cells, as determined after 48, 72, and 144 h of incubation.

For all antiviral/cytotoxicity assays, we used in-house synthesized diphyllin **1** (Scheme 1, Figure 1A), whose biological activities were compared with the commercially synthesized compound purchased from Apigenex (Prague, Czech Republic) (Figure S1). To compare the biological activities of both compounds, we performed a viral titer reduction assay; virus and compound were added simultaneously to Vero cells and cultivated for 48 h at 37 °C under 5% CO_2_ (see Section 2.11. simultaneous treatment). The diphyllinoside known as diphyllin-4-β-D-glucopyranoside (cleistanthin B) **8** was obtained from WuXi AppTec (Tianjin, China). All compounds were solubilized in dimethyl sulfoxide (DMSO) as 10 mM stock solutions.

### 2.10. Cytotoxicity Assays

To determine the cytotoxicities of diphyllin **1** and diphyllinoside cleistanthin B 8, we used multiple cells/cell lines of neural and extraneural origin. Vero, PS, UKF-NB-4, Huh-7, or HBCA cells were seeded in 96-well microtitration plates (2 × 10^4^ cells/well), and incubated for 24 h at 37 °C. After incubation, diphyllin **1** or cleistanthin B **8** were added to the cells (0 to 100 µM) then treated cells were further cultivated for 48 h at 37 °C. Cytotoxicity of diphyllin **1** for BHK-21 cells was measured after 72 h cultivation. In order to evaluate Vero cell cytotoxicities from exposure to diphyllin **1**, the cells were seeded in 96-well microtitration plates (2 × 10^4^ cells/well), incubated for 24 h at 37 °C under 5% CO_2_, and then diphyllin **1** or cleistanthin B **8** (0 to 100 µM) was added. The treated cells were further cultivated for 48, 72, and 144 h at 37 °C under 5% CO_2_. Cytotoxicity of bafilomycin A1 (0 to 200 µM) was assayed in Vero cells after a 48-h incubation at 37 °C under 5% CO_2_. The cytotoxicity measured in terms of cell viability was determined with Cell Counting Kit-8 (Dojindo Molecular Technologies, Munich, Germany), according to the manufacturer’s instructions. The respective concentrations of each compound under investigation that reduced cell viability by 50% (CC_50_ values) were determined.

### 2.11. Antiviral Assays

Viral titre reduction assays were performed to determine the sensitivity of the tested viruses to diphyllin **1** in cell culture. Vero cells were used for the evaluation of the antiviral efficacy of diphyllin **1** against TBEV, WNV, ZIKV, RVFV, and HSV-1. Host cells were seeded in 96-well plates (approximately 2 × 10^4^ cells per well) and incubated for 24 h at 37 °C under 5% CO_2_ to form a confluent monolayer.

For each virus, two independent experiments were performed in triplicate in each case. In each independent experiment, infected Vero cells were treated with drug at three different times, as follows: (i) 2 h pre-treatment assays—media (200 µL) with diphyllin **1** (0 to 25 µM) (2-fold dilution, three wells per concentration) were added to cell monolayers 2 h prior to infection. After 2 h incubation at 37 °C under 5% CO_2_, media were aspirated and replaced with fresh compound-containing media (200 µL) in the same concentration range, then inoculated with the appropriate virus at an MOI of 0.1. Cells were further incubated for 48 h at 37 °C under 5% CO_2_; (ii) simultaneous treatment assays—media containing diphyllin **1** (0 to 25 µM) were inoculated with the appropriate virus (MOI of 0.1) and added to cells that were then incubated for 48 h at 37 °C under 5% CO_2_; (iii) 2 h post-treatment assays—Vero cells were first infected with the appropriate virus (MOI of 0.1), and 2 h after infection (the time needed for virus adsorption and internalization), media containing diphyllin **1** (0 to 25 µM) were added to infected cells that were then incubated for 48 h at 37 °C under 5% CO_2_. 

Following incubation, media were collected and viral titers were determined by plaque assay (see below) to construct dose-dependent inhibition curves. Since the greatest inhibitory effects were observed in 2 h pre-treatment assays, then titer values determined from these assays were also used to estimate 50% effective concentration (EC_50_) values. The antiviral activity of cleistanthin B **8** was determined only for TBEV in Vero cells, using the same protocol as described above. Media were collected 48 h after infection, and TBEV titers were estimated using plaque assays once again. 

For anti-RABV assays, a suspension of BHK-21 cells (5 × 10^4^ cells per a well) was incubated with diphyllin **1** (0 to 25 µM) and with RABV (10^6.58^ TCID_50_/mL) at 37 °C under 5% CO_2_ for 40 min, mixing every 10 min. Following incubation, the RABV-infected cells were seeded on 96 well plates and cultivated for 72 h at 37 °C under 5% CO_2_ (a longer incubation time was used compared with other viruses tested since RABV does not typically generate sufficiently high titers when incubated for 48 h). Thereafter, media samples were collected, and the RABV RNA was quantified using quantitative real-time PCR (RT-qPCR). Two independent experiments were performed in triplicate (as above).

Antiviral assays with bafilomycin A1 were performed with Vero cells seeded in 96-well plates (2 × 10^4^ cells per well) and incubated for 24 h at 37 °C under 5% CO_2_ to form a confluent monolayer. Media containing bafilomycin A1 (0 to 100 nM) were simultaneously infected with ZIKV (strain Paraiba_01) or HSV-1 (strain MacIntyre) (MOI of 0.1) and added to cells that were then incubated for 48 h at 37 °C under 5% CO_2_. Viral titers were determined by plaque assays.

### 2.12. Plaque Assay

To quantifiy the viral titres for ZIKV, WNV, RVFV, and HSV-1, plaque assays were performed using Vero cells. For TBEV viral titer determination, we performed PS cell-based plaque assays. In both cases, a modified protocol was used as originally developed by De Madrid and Porterfield [29]. Briefly, 10-fold dilutions of the virus were prepared in 24-well tissue culture plates, and the cells were added to each well (0.6–1.5 × 10^5^ cells/well). After 4 h incubation, the suspension was overlaid with 3% (*w/v*) carboxymethylcellulose in DMEM (for Vero cells) or L15 (for PS cells). Following day 5 of incubation at 37 °C and 5% CO_2_ (DMEM) or in a normal atmosphere (L15), the infected plates were washed with phosphate-buffered saline, and the cell monolayers were stained with naphthalene black. The virus titer was expressed as plaque-forming units (PFU)/mL.

### 2.13. Quantitative Reverse Transcription PCR (RT-qPCR)

RABV RNA was isolated from growth media supernatants using the QIAmpViral RNA mini kit (Qiagen, Germantown, MD, USA) following the manufacturer’s instructions. RT-qPCR measurements were performed on the LightCycler 480 II in a 96-well plate block (Roche, Basel, Switzerland) using the Advanced kit for Tick-borne encephalitis (Genesig, Germantown, MD, USA) and Lyophilised OneStep qRT-PCR (Oasig) according to manufacturer’s instructions. RABV RNA copy numbers/mL was calculated from calibration curves based on standards provided by the manufacturer (Genesig, Germantown, MD, USA).

### 2.14. Immunofluorescence Staining

In order to measure the compound-induced inhibition of viral antigen expression, a cell-based immunostaining assay for TBEV, WNV, ZIKV, RVFV, RABV, and HSV-1 was performed. Vero cells were used for the cultivation of TBEV, WNV, ZIKV, RVFV, and HSV-1 and BHK-1 cells for RABV cultivation. 

Vero cells were seeded onto 96-well microtitration plates, cultivated for 24 h, and then treated with diphyllin **1** (0 to 25 μM) (2-fold dilution, three wells per concentration) for 2 h. Thereafter, cell monolayers were infected with the appropriate virus (MOI of 0.1) and cultured for 48 h at 37 °C under 5% CO_2_. After incubation, the cells were fixed with cold acetone:methanol (1:1, *v/v*) and blocked with 10% fetal bovine serum. For flavivirus immunostaining (i.e., TBEV, WNV, and ZIKV), cells were incubated with a mouse monoclonal antibody targeting the flavivirus group surface antigen (protein E) (1:250; antibody clone D1-4G2-4-15; Sigma-Aldrich, Prague, Czech Republic). Anti-Herpes Simplex Virus Type 1/2 antibody (1:250; antibody clone M4091313) and Anti-Nucleoprotein antibody (1:250; AA 1-245, UniProt: P21700) were obtained from Antibodies-online GmbH (Aachen, Germany) and were used for the immunostaining of HSV-1 and RVFV, respectively. Subsequently, cells were labeled with an anti-mouse goat secondary antibody conjugated with fluorescein isothiocyanate (FITC; 1:500; Sigma-Aldrich, Prague, Czech Republic) following 1 h incubation at 37 °C. Cells were then counterstained with 4′,6-diamidino-2-phenylindole (DAPI; 1 μg/mL; Sigma-Aldrich, Prague, Czech Republic) to visualize cell nuclei. Fluorescence signals were recorded using an Olympus IX71 epifluorescence microscope (Olympus Corporation, Tokyo, Japan).

For the immunostaining of RABV-infected cells, a suspension of BHK-21 cells (5 × 10^4^ cells per well) was incubated with diphyllin **1** (0 to 25 µM) (2-fold dilution, three wells per concentration) and RABV (10^6.58^ TCID_50_/mL) at 37 °C for 40 min, mixing every 10 min. Following incubation, the RABV-infected cells were seeded on a 96 well plate and cultivated for 72 h. After fixation with cold acetone–methanol (1:1, *v/v*) and blocking with 10% fetal bovine serum, the cells were incubated with an Anti-Rabies-Specific monoclonal antibody (1:250; Art.No. PA1202; Enzo Life Science, NY, USA) labeled with FITC (green). As above, cell nuclei were counterstained with DAPI (1 μg/mL), and fluorescence signals were recorded, also as described above.

### 2.15. Analysis of Endosomal Acidification Effects 

Vero cells (1 × 10^6^ cells/well) were cultured in a 6-well microtiter plate for 24 h. Then, the growth medium was aspirated and replaced with a fresh medium containing diphyllin **1** (100 µM) or bafilomycin A1 (100 nM) at 37 °C for 20 min. Bafilomycin A1 was used as a positive control, and DMSO 1% (*v/v*) was applied as a negative control. Acridine orange dye (1 μg/mL; Sigma-Aldrich) was added to each well and incubated at 37 °C for 10 min. Fluorescence signals were recorded using an Olympus IX71 epifluorescence microscope (Olympus Corporation, Tokyo, Japan). The red and green fluorescence signals were acquired using Texas Red and FITC excitation filters, respectively.

### 2.16. Mechanistic Analysis of Diphyllin-Mediated Antiviral Effects

Vero cells were seeded in 6-well plates and incubated for 24 h at 37 °C under 5% CO_2_ until confluent. Thereafter, a virus inoculum (10^6^ PFU/mL, ZIKV strain Paraiba_01 or HSV-1 strain MacIntyre) and diphyllin **1** (100 µM) or bafilomycin A1 (100 nM) were added to the cells and incubated for 3 h. Following incubation, Vero cells were washed with culture medium to remove the compounds, and the non-adsorbed virus and fresh medium with 4% (*w/v*) carboxymethylcellulose were added to the cells. After further incubation for 72 (with HSV-1) or 120 h (with ZIKV), Vero cell monolayers were stained with naphthalene black, and the number of plaques was counted. Control cells were not washed, and the compounds and virus were retained in cell culture until the end of the experiment. Following a 3 h incubation, 4% (*w/v*) carboxymethylcellulose was added to the cells, and the cells were incubated for 72 or 120 h, as described above. Then, the monolayers were stained with naphthalene black, and the number of plaques was counted.

## 3. Results

### 3.1. Synthesis of Diphyllin ***1***

Our revised, updated, and improved synthesis of diphyllin **1** was devised with reference to previous literature [24,30,31] and is illustrated (Scheme 1). Although diphyllin **1** is a known compound, the published synthetic routes proved difficult to implement in our hands, and key intermediates were incompletely characterized throughout. Here we have resolved these difficulties and omissions to describe a robust, scalable, fully characterized synthesis. Exceptionally, our synthetic route began with the formation of benzo-[d]-[1,3]dioxole-5-carbaldehyde **2** from 3,4-dihydroxybenzaldehyde. In parallel, 2-(2-bromo-4,5-dimethoxyphenyl-[1,3]dithiane **3** was prepared by combination of 2-bromo-4,5-dimethoxybenzaldehyde with 1,3-propanedithiol. Both **2** and **3** were coupled efficiently to give alcohol **4** following initial transmetallation of **3** with n-butyl lithium. Oxidation of **4** with MnO_2_ gave rise to benzophenone intermediate **5** as a crystalline compound. Thereafter, the formation of key intermediate **6**, through intermolecular Michael-addition of 2(5H)-furanone to **5** with subsequent ring closure, proved the most challenging step. Reagents previously described in the literature for this purpose [24], such as lithium diisopropylamine (LDA), resulted in multiple product formation. In the event, key intermediate **6** was only prepared successfully through judicious use of the reagent LiHMDS in dry THF at −65 °C for 48 h. The use of this reagent also enabled the recovery of unreacted starting materials by column chromatography for reuse in subsequent repeat reactions. Thereafter, dithiane deprotection of key intermediate **6** was then carried out in the standard way, and the resulting compound **7** was aromatized with p-toluenesulfonic acid to give diphyllin **1** (Scheme 1). This was obtained in excellent purity but found to be rather light-sensitive and subject to surface coloration with detrimental effects on antivirus activities. Therefore, once prepared, diphyllin **1** samples were customarily stored in the dark in the fridge (2–8 °C), until further required. The elemental analysis of diphyllin **1**, C_21_H_16_O_7_ (380.35) was calculated as C, 66.31; H, 4.24., and found to be C, 66.45 ± 0.10; H, 4.56 ± 0.02. These compare to values of C, 66.73 ± 0.07; H, 4.35 ± 0.01 measured from a small control sample of diphyllin **1** provided commercially (Apigenex, Prague, Czech Republic). 

A functional comparison between our synthetic diphyllin **1** and the commercial product was carried out using an anti-TBEV antiviral assay. Both compounds (0–25 µM), when cultured in TBEV-infected Vero cells, resulted in sigmoidal growth curves of similar shapes and slopes with EC_50_ values of 1.04 µM and 1.24 µM for the in house and commercially synthesized compounds, respectively. This indicates that the biological activities of both compounds were essentially identical (Figure S1). 

### 3.2. Cytotoxicity of Diphyllin ***1*** and Cleistanthin B ***8***

The cytotoxicities of diphyllin **1** and diphyllinoside cleistanthin B **8** were determined with respect to five different immortalized cell lines (i.e., Vero, PS, BHK-21, UKF-NB-4, and Huh-7 cells) and primary HBCA cells. These cells were used since they are often used for in vitro cultivation and for antiviral assays with numerous medically important viruses. The UKF-NB-4 and HBCA cells are of neural origin and so are highly suitable for in vitro multiplication and antiviral studies involving neurotropic viruses. Diphyllin **1** and cleistanthin B **8** were found to be substantially cytotoxic to UKF-NB-4, Huh-7, and HBCA cells 48 h post-administration, in a dose-dependent manner (Figure 1C,D). 

The CC_50_ values for UKF-NB-4, Huh-7, and HBCA cells were calculated to be 11.13, 1.89, and 3.39 µM, respectively (for diphyllin **1**), and 6.17, 3.96, and 2.08 µM, respectively (for cleistanthin B **8**) (Table 1). Diphyllin **1** and cleistanthin B **8** were found to be slightly cytotoxic for PS cells (CC_50_ values of 92.44 and 78.86 µM, respectively) (Figure 1C,D, Table 1). Only Vero cells and BHK-21 cells proved essentially robust to diphyllin **1** and cleistanthin B **8** administration up to a concentration threshold of 100 µM. The prolonged incubation of Vero cells with diphyllin **1** and cleistanthin B **8** up to 72 h did not have substantial cytotoxic effects. However, the 144 h incubation of diphyllin **1** with Vero cells did result in a significant decrease in Vero cell viability (CC_50_ of 18.19 µM). Interestingly, cleistanthin B **8** was less cytotoxic, when incubated for 144 h with Vero cells (CC_50_ of 80.73 µM) (Figure 1E,F; Table 1). Based on the observed cytotoxicity profiles of the cell lines studies, Vero cells were used for all antiviral assays with TBEV, WNV, ZIKV, RVFV, and HSV-1, whereas BHK-1 cells were used for anti-RABV assays. 

### 3.3. Antiviral Activity of Diphyllin ***1*** and Cleistanthin B ***8*** against Tick- and Mosquito-Borne Flaviviruses

**Figure 2:**
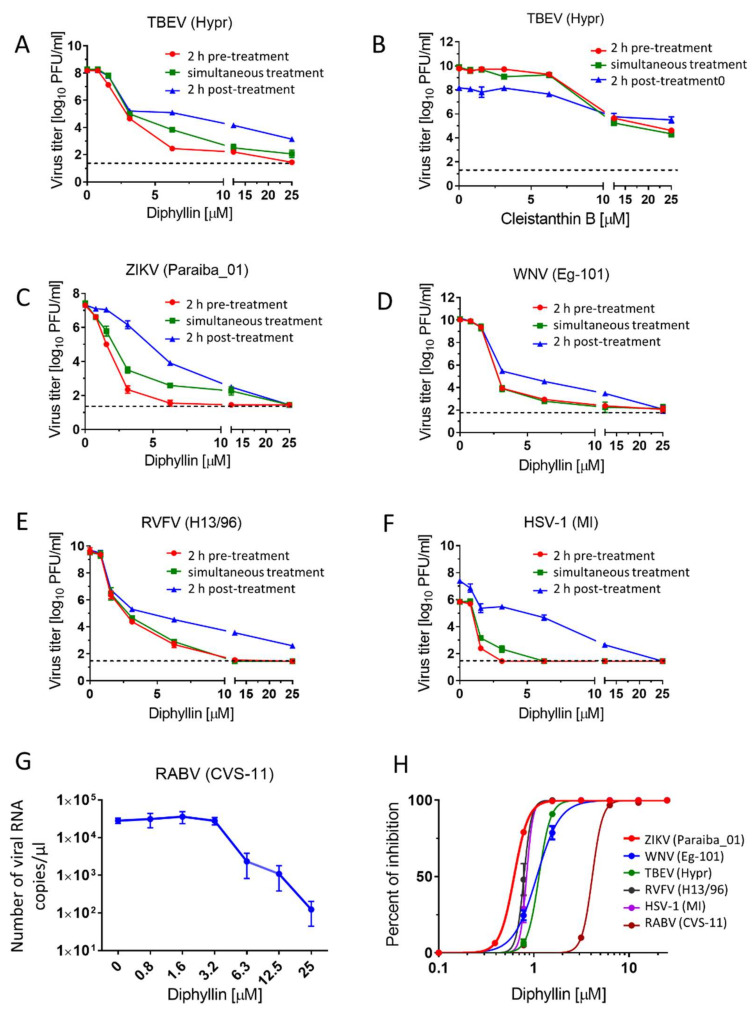
Antiviral activities of diphyllin **1** and cleistanthin B **8**. (**A**,**C**–**F**) Anti-antiviral activities of diphyllin **1**. Vero cell monolayers were treated with diphyllin **1** (0 to 25 µM) either 2 h prior to TBEV (**A**), ZIKV (**C**), WNV (**D**), RVFV (**E**), and HSV-1 (**F**) infection (at a MOI of 0.1) (2 h pre-treatment), simultaneously with virus infection (simultaneous treatment), or 2 h after infection (2 h post-treatment). The infected cells were then incubated for 48 h, after which cell media were collected, and viral titers were determined using a plaque assay and expressed as PFU/mL. (**B**) Anti-TBEV activity of cleistanthin B **8** (0 to 25 µM). The experimental procedure was the same as in (**A**). (**G**) Anti-RABV activity of diphyllin **1**. A suspension of BHK-21 cells was incubated with diphyllin **1** (0 to 25 µM) and with RABV (10^6.58^ TCID_50_/mL) at 37 °C for 40 mins. Following incubation, the RABV-infected cells were seeded on 96 well plates and cultivated for 72 h then, media were collected, and the RABV RNA was quantified by RT-qPCR. (**H**) Inhibition curves for diphyllin **1** were constructed from the viral titer values (2 h pre-treatment) in order to estimate EC_50_ values for the indicated viruses. The mean titers from three biological replicates of two independent experiments are shown, and error bars indicate standard errors of the mean (*n* = 3). The horizontal dashed line indicates the minimum detectable threshold of 1.44 log_10_ PFU/mL. MI, MacIntyre.

The antiviral activities of diphyllin **1** and cleistanthin B **8** were first evaluated against a representative of the tick-transmitted arboviruses, namely TBEV (Hypr strain). For the anti-TBEV assays, we used three different treatment regimens, differing in the time of drug addition to cell monolayers (2 h pre-treatment, simultaneous treatment, and 2 h post-treatment, see above). Diphyllin **1** showed strong antiviral activities against TBEV in all three treatment regimens (Figure 2A). However, antiviral activities were most pronounced when diphyllin **1** was administered 2 h prior to infection (2 h pre-treatment, EC_50_ of 0.85 µM). The differences in viral titer decrease between individual treatment regiments were obvious mostly at high compound concentrations and did not have substantial effects on the EC_50_ values. Using the 2 h pre-treatment, diphyllin **1** was able to almost completely eliminate TBEV replication in Vero cell culture at concentrations between 12.5 and 25 µM (Figure 2A,H, Table 2). By contrast, although the peak anti-TBEV activity of cleistanthin B **8** was observed in pre-treatment assays, the level of activity was almost 6.3-fold lower than observed with diphyllin **1** (Figure 2B, Table 2). Owing to this low antiviral activity compared to diphyllin **1** and only a comparable cytotoxicity profile, cleistanthin B **8** was excluded from further antiviral analyses. 

Thereafter, diphyllin **1** was evaluated against another medically important emerging flavivirus, namely WNV (strain Eg-101), a representative of the mosquito-borne flaviviruses. ZIKV (strain Paraiba_01, from Brasil), another key member of the *Flaviviridae* family, which is transmitted by mosquitoes, was also studied here but as a positive control, given that ZIKV was recently found susceptible to a diphyllin analog, diphyllinoside patentiflorin A [13]. As with TBEV, Vero cells were treated with diphyllin **1** 2 h prior to infection (2 h pre-treatment), simultaneously with infection (simultaneous treatment), and 2 h after infection (2 h post-treatment) (Figure 2C,D). We observed the highest antiviral activities when diphyllin **1** was administered 2 h pre-treatment (EC_50_ values of 0.78 and 0.54 µM for WNV and ZIKV, respectively). Furthermore, using 2 h pre-treatment, diphyllin **1** was able to completely suppress WNV and ZIKV replication in Vero cell culture at concentrations between 12.5 and 25 µM (Figure 2C,D, Table 2). 

### 3.4. Antiviral Activity of Diphyllin ***1*** against RVFV, RABV, and HSV-1

Out of curiosity, the antiviral activities of diphyllin **1** were further tested against one representative each of enveloped RNA viruses from the *Phenuiviridae* family (RVFV, strain H13/96) and the *Rhabdoviridae* family (RABV, strain CVS-11), plus one representative of the enveloped DNA viruses from the *Herpesviridae* family (HSV-1, strain MacIntyre). The sensitivity of RVFV and HSV-1 to diphyllin **1** was evaluated with Vero cell infection, whereas anti-RABV assays were performed with BHK-21 cells. Both cell lines were robust to diphyllin **1** administration up to a concentration threshold of 25 µM (see above). In these cases, diphyllin **1** also showed strong antiviral activities against RVFV, although once again, antiviral activities were most pronounced when diphyllin **1** was administered 2 h prior to infection (2 h pre-treatment, EC_50_ of 0.46 µM) with complete suppression of viral replication between 12.5 and 25 µM. Very similar results were observed in HSV-1 (2 h pre-treatment, EC_50_ of 0.59 µM) with complete viral suppression starting at 2.5 µM (Figure 2E,F,H, Table 2). The ability of diphyllin **1** to inhibit RABV replication in cell culture was studied by quantification of viral genome RNA in cell culture media using RT-qPCR 72 h post-infection. Diphyllin **1** was shown to be a strong inhibitor of RABV RNA synthesis (EC_50_ of 6.33 µM), resulting in a 10^2^ drop in RABV RNA copies/µL compared with RABV-infected control BHK-21 cells (Figure 2G,H; Table 2). Higher EC_50_ values compared to other viruses are largely due to different methodologies for determining viral titer in anti-RABV assays (RT-qPCR instead of plaque assay, different conditions during infection, etc.).

### 3.5. Inhibition of Expression of Virus Antigens with Diphyllin ***1***

**Figure 3:**
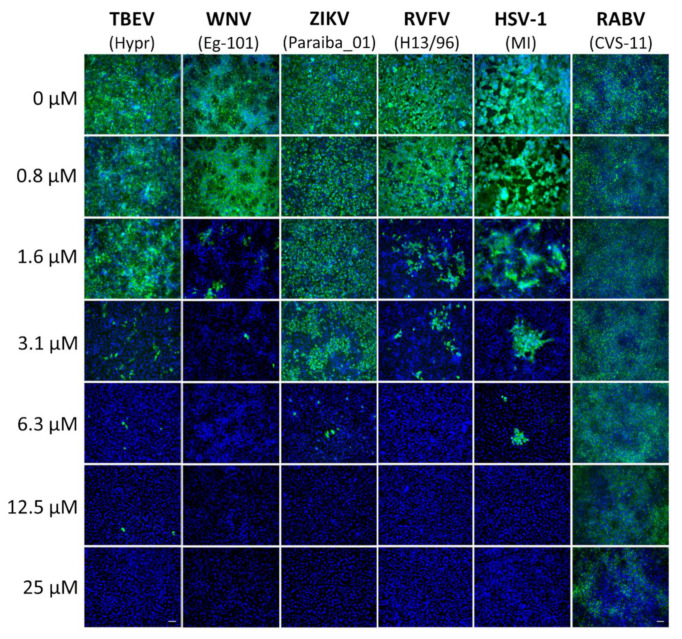
Inhibition of viral surface antigen expression by diphyllin **1**. For immunostaining of TBEV-, WNV-, ZIKV-, RVFV-, and HSV-1-infected cells, Vero cell monolayers were treated with diphyllin **1** (at the indicated concentrations) and infected with the appropriate virus (MOI of 0.1). Cells were fixed on slides 48 h after infection, stained with viral antigen-specific antibody (primary antibody), and an anti-mouse goat antibody conjugated with FITC (green) (secondary antibody), plus counterstained with DAPI (blue). For immunostaining of RABV-infected cells, a suspension of BHK-21 cells was infected with RABV (10^6.58^ TCID_50_/mL) at 37 °C for 40 min and incubated with diphyllin **1** (0 to 25 µM), mixing every 10 min. Following incubation, the RABV-infected cells were seeded on 96-well plates and cultivated for 72 h. After fixation and blocking, the cells were incubated with an antirabies-specific antibody labeled with FITC (green), and the cell nuclei were counterstained with DAPI (blue). MI, MacIntyre. Scale bars, 50 µm.

Diphyllin **1** dose-dependent antiviral effects were confirmed by immunofluorescent staining, which detects expression of viral antigens of TBEV, WNV, ZIKV, RVFV, and HSV-1 in Vero cells in vitro as a marker of viral infectivity and replication. Compared with control cells, a clear decrease in fluorescence signal was observed with increasing diphyllin **1** concentrations, monitored 48 h post-infection. In line with the viral titer data shown above (Figure 2A,C–H, Table 2), viral surface antigen expression in Vero cell culture was eliminated at administered diphyllin **1** concentrations between 12.5 and 25 µM (Figure 3). The effect of diphyllin **1** administration on the expression of RABV surface antigens was the least pronounced of all viruses tested, although the obvious decrease in fluorescence signal intensity was still observed at diphyllin **1** concentrations between 12.5 and 25 µM (Figure 3), in keeping with diphyllin **1** mediated reductions in RABV RNA copies/µL. 

### 3.6. Mechanism of Action of Diphyllin **1**

**Figure 4:**
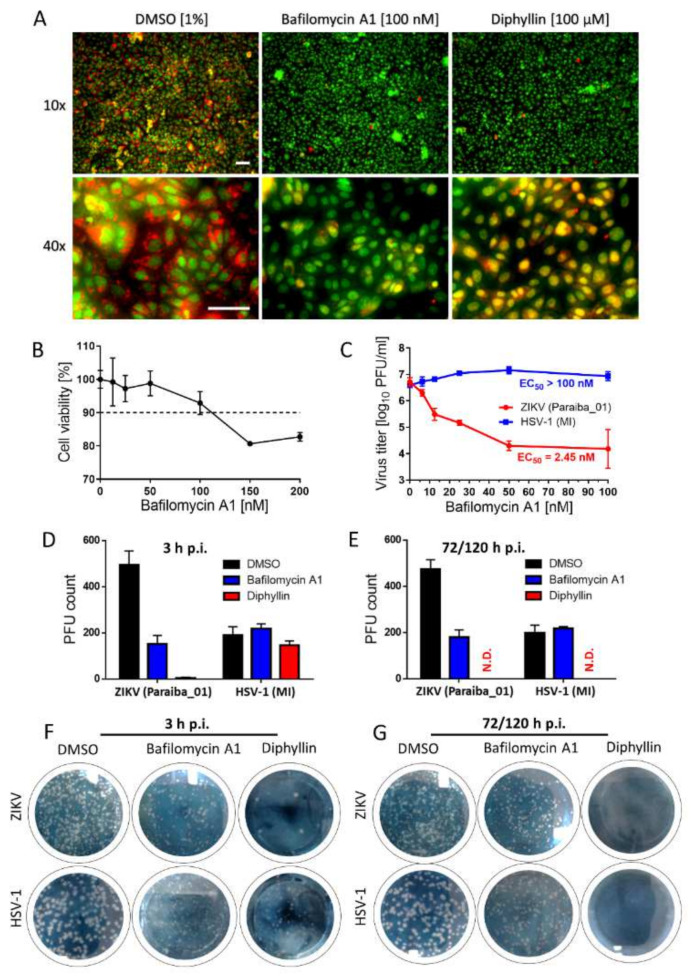
Diphyllin **1** mechanism of action assessment and comparison of biological activity of diphyllin **1** with that of bafilomycin A1. (**A**) Analysis of endosomal acidification with acridine orange labeling. Vero cells cultured in 96-well microtiter plates were treated with diphyllin **1**, bafilomycin A1, or DMSO (1%, *v/v*) (controls) at the indicated concentrations at 37 °C for 20 min and then incubated with acridine orange dye (1 μg/mL). Scale bars, 50 µm. (**B**) Cytotoxicity of bafilomycin A1 for Vero cells in the indicated concentration range. The cells were seeded in 96-well plates for 24 h, then treated with bafilomycin A1 and incubated for 48 h. (**C**) Anti-ZIKV and anti-HSV-1 activities of bafilomycin A1 in Vero cells. Vero cell monolayers were treated with bafilomycin A1 and simultaneously inoculated with ZIKV or HSV-1 (MOI of 0.1). The infected cells were then incubated for 48 h, after which cell media were collected, and viral titers were determined using a plaque assay. (**D**) Anti-ZIKV and anti-HSV-1 activities of diphyllin **1** (100 µM) and bafilomycin A1 (100 nM) in Vero cells measured after 3 h p.i. DMSO-treated cells were used as controls. Vero cell monolayers inoculated with the indicated viruses and diphyllin **1** (100 µM), bafilomycin A1 (100 nM), or DMSO (1%, *v/v*) were added to the cells for 3 h at 37 °C. After extensive washing, fresh medium with 4% (*w/v)* carboxymethylcellulose was added to the cell monolayers. After 72 h (for HSV-1) or 120 h (for ZIKV), the cells were stained with naphthalene black, and the number of plaques was determined. (**E**) Control cells were inoculated with the appropriate viruses and incubated with the compounds for 72 or 120 h for HSV-1 and ZIKV, respectively. Then, the cells were stained with naphthalene black, and the number of plaques was determined. (**F**,**G**) ZIKV and HSV-1-infected Vero cell monolayers in 6-well plates treated with the indicated compounds. The experimental conditions were described above, see (**D**,**E**). The horizontal dashed line represents 90% threshold for compound cytotoxicity assessment. MI: MacIntyre; N.D.: not detected.

Diphyllin **1,** as a known V-ATPase inhibitor, should act as a blocker of endosomal acidification in Vero cells. This was verified by comparing the effect on cells of diphyllin **1** (at 100 μM), bafilomycin A1 (at 100 nM) (a well-documented endosome acidification blocker that also inhibits V-ATPases [13,17]), and DMSO (1%, *v/v*, used as negative control), all in the presence of acridine orange dye. After cell uptake, this dye enters endosome compartments, and those that are at neutral pH appear green under the fluorescence microscope, while those that have become mildly acidic appear red owing to dye protonation. According to our data, control cells treated with DMSO exhibited an extensive red fluorescent signal indicative of a high number of acidic endosomes (Figure 4A). By contrast, in cells treated with diphyllin **1** (100 µM), red fluorescence intensities appear much reduced, indicative of a much lower number of acidic endosomes and an increased number of neutral endosomes. This situation is all the more obvious in cells treated with bafilomycin A1 (100 nM). Taken together, these data confirm that diphyllin **1** is able to inhibit endosomal acidification in Vero cells, consistent with its known role as a V-ATPase inhibitor, although bafilomycin A1 is considerably more potent in this regard. Please note that cell nuclei were clearly visible in acridine orange-stained Vero cells too, since acridine orange is a strong nucleic acid binder that emits green fluorescence when bound to dsDNA (Figure 4A).

Following the above, cytotoxicity studies were performed with bafilomycin A1 to establish that concentrations of ≥150 nM reduced Vero cell viabilities to approximately 80% compared with control cells (Figure 4B). Therefore subsequent functional bafilomycin A1-based studies were performed with a concentration maximum of 100 nM only, even though concentrations in the 100–200 nM were widely used during previous bafilomycin A1-based studies [13,22]. In functional studies, bafilomycin A1 was shown to affect a dose-dependent reduction in ZIKV (strain Paraiba_01) virus titers 48 h post-infection of Vero cells with virus and the addition of bafilomycin A1 (Figure 4C). The EC_50_ value was found to be 2.44 nM, suggesting that bafilomycin A1 is 10^3^-fold more potent inhibitor of the ZIKV replication cycle than diphyllin **1**. By contrast, bafilomycin A1 had no effect on the corresponding HSV-1 (strain MacIntyre) virus titers (Figure 4C) up to 100 nM. 

Additional anti-ZIKV and anti-HSV-1 studies were then performed with Vero cells subject to simultaneous treatment with virus and bafilomycin A1 (100 nM) or diphyllin **1** (100 μM) for 3 h after which plaque numbers were counted at 72 h p.i. (with HSV-1) and 120 h p.i. (with ZIKV). We used a longer incubation period (120 h) for the ZIKV-based experiment, as such incubation time was needed for the formation of clear, well observable plaques. Control (virus-infected, mock-treated) cells were incubated with the compounds for the whole experimental period, i.e., for 72/120 h p.i. These data at 3 h p.i. (virus-infected, drug-treated cells, Figure 4D,F) and 72/120 h p.i. (control cells, Figure 4E,G) reveal that ZIKV was susceptible to inhibition by both drugs at 3 h p.i. and 120 h p.i. In contrast, HSV-1 was not susceptible to bafilomycin A1 treatment at 3 h p.i. or 72 h p.i. Moreover, HSV-1 was not susceptible to diphyllin **1** treatment at 3 h p.i., but was highly susceptible to diphyllin **1** at 72 h p.i. These data were not only consistent with the anti-ZIKV and anti-HSV-1 properties of diphyllin **1** measured previously (EC_50_ values were 0.54 and 0.59 µM, respectively—see Table 2) but also suggested that the anti-HSV-1 mechanisms of action of diphyllin **1** target later stages of the HSV-1 replication cycle (i.e., replication/assembly phases) rather than the early stages (i.e., cell entry/fusion phases).

## 4. Discussion

Since diphyllin **1** is very often found in plants in the form of glycosides, initial efforts were made to cross-compare biological activities of diphyllin **1** with the glycoside analogue cleistanthin B **8**. Prior to full antiviral studies, the cytotyoxicity profiles of diphyllin **1** and cleistanthin B **8** were studied in a range of cells lines. Vero and BHK-1 cells were resistant to the administration of both compounds. However, both compounds were cytotoxic for PS, UKF-NB-5, and Huh-7 cell lines, and also for primary human astrocytes HBCA (Figure 1, Table 1). Indeed, diphyllin **1** has been found cytotoxic to many other cell lines besides such as Mardin-Darby canine kidney cells (MDCK, CC_50_ of 3.48 µM) [22], human lung carcinoma cells (A549, CC_50_ of 24.01 µM) [22], feline macrophage-like cells (fcwf-4, CC_50_ of 5.99 µM) [17], and rabbit lung cells (RL-33 CC_50_, of 63 µg/mL) [25]. Given the anticancer capabilities of diphyllin **1** and analogs [7,8,32,33,34,35,36], such activities against multiple immortalized cell lines should not perhaps be so surprising. Accordingly, diphyllin is similar to other V-ATPase blockers, which were recently described to have significant anticancer potencies indicative of general anticancer drugs. These include, plecomacrolide antibiotics, classical V-ATPase inhibitors such as concanamycin and bafilomycin A1, benzolactone enamides (apicularens, lobatamides, oximidines, and cruentaren), archazolid, indolyls, and late-generation V-ATPase inhibitors, such as FR202126, 3-bromopyruvate, or tributyltin chloride [37]. Importantly, the antiviral effects of diphyllin **1** are observed at considerably lower (non-cytotoxic) concentrations.

Here we demonstrate that diphyllin **1** acts as a sub-micromolar or low-micromolar inhibitor of multiple enveloped RNA viruses from the *Flaviviridae, Phenuiviridae,* and *Rhabdoviridae* families (Figure 2, Table 2), while cleistanthin B **8** displayed significantly lower antiviral effects compared with those for diphyllin **1** and was not therefore studied further here. In comparison, a diphyllin **1** and the diphyllin analog patentiflorin A have been shown previously to block ZIKV replication in vitro and prevented mortality in a rodent in vivo model of ZIKV infection as alluded to above [13]. In contrast with our study, the ZIKV infectivity in diphyllin **1**-treated cells was evaluated using immunofluorescence staining-based method demonstrating the anti-ZIKV effect of diphyllin **1** even in nanomolar concentrations [13]. Furthermore, analogs patentiflorin A and justiprocumin B, were found to display nanomolar activity against four clinical HIV-1 isolates in human peripheral blood mononuclear cells (PBMC) cells [23,24] as well broad spectrum nanomolar inhibition of HIV-1 strains resistant to several reverse transcriptase inhibitors [24]. In addition, diphyllin **1** itself has been shown to inhibit the replication of feline infectious peritonitis virus (FIPV), a member of the *Coronaviridae* family, in fcwf-4 cells. Furthermore, diphyllin **1** has also been shown to be potent in vitro against influenza virus in MDCK cells [22]. Some other diphyllin derivatives (e.g., justicidin A and B, and some others) have also been found active against vesicular stomatitis virus (VSV) when tested in RL-33 cells [25]. Therefore, taken together with our data here, there can be no doubt that diphyllin **1** is a potentially useful broad antiviral agent through its broad cellular cytotoxicity profile suggests that this antiviral agent would be best targeted to sites of viral infection by means of drug delivery nanoparticles and other nanomedicine approaches [17]. 

In mechanistic terms, the success of diphyllin **1** as a broad antiviral agent, as demonstrated here using complementary in vitro virus infection models, might seem somewhat paradoxical. Given that diphyllin **1** is a known V-ATPase inhibitor and hence inhibitor of endosome acidification, then this mechanism of action should account for the inhibition of the replication cycle of viruses that enter host cells by endocytosis followed by acid pH-induced membrane fusion [13,17,22]. This is certainly true of *Flaviviridae, Bunayviridae,* and *Rhabdoviridae* families. All these viruses enter host cells through endocytosis and require low pH-induced fusion of viral envelope with endosome membrane to continue their replication cycles [16]. Consistent with this argument, our data here indicate clearly that the mechanism of action of diphyllin **1** against ZIKV, a member of the Flaviviridae family, takes place during the early phases of the virus replication cycle on a time scale consistent with a mechanism of action involving V-ATPase inhibition and the direct modulation of endosome acidification to abrogate the virus replication cycle, all in keeping with the known mechanism of ZIKV host cell entry by endocytosis [13].

On the other hand, diphyllin **1** also exhibits sub-micromolar antiviral potency against HSV-1, a virus that enters cells by plasma membrane interactions and cell surface fusion (not via endocytosis) at neutral pH (with no need for V-ATPase activity) [16]. Hence, our observed diphillin-mediated anti-HSV-1 effects cannot be based on the inhibition of HSV-1 early cell entry but rather on the suppression of other, later stages of the HSV-1 replication cycle. Accordingly, diphillin **1**, in marked contrast to bespoke V-ATPase inhibitors such as bafilomycin A1, must be able to employ additional, alternative antiviral mechanisms of action to abrogate viral replication cycles at different stages as appropriate. Indeed, besides being a V-ATPase inhibitor, diphyllin **1** and diphyllinosides have also been shown to target topoisomerase IIα [38], to induce apoptosis and protective autophagy through reactive oxygen species production [8], block voltage-gated K^+^ channels in mouse neuroblastoma cells [39], stimulate interferon-γ production [40], reduce nitric oxide levels [41], and modulate TNF-α, and IL-12 production in mouse macrophages [12]. Hence, we speculate that any or even some of these alternative capabilities might be used to suppress viral infection cycles that do not begin with endocytosis followed by acid pH-induced fusion of viral and endosomal membranes. 

Besides diphyllin **1** and bafilomycin A1, broad-spectrum antiviral activity was previously demonstrated also for other V-ATPase blockers, such as saliphenylhalamide (SaliPhe), tenatoprazole, esomeprazole, and ammonium chloride, to name a few. The expected molecular target for SaliPhe is a binding site of the proton translocation domain of cellular V-ATPase; this compound can effectively inhibit several wild types of influenza viruses [42]. A similar mode of action was also considered for ammonium chloride, which showed inhibitory activity against human rhinoviruses [43]. In addition, ammonium chloride blocked infection of human papillomavirus (PV) type 16 by preventing the transport of PV viral particles from early endosomes to caveosomes [44]. Multiple modes of antiviral action were also described for tenatoprazole and esomeprazole. In addition to proton pump blocking, these compounds were observed to inhibit the budding of some enveloped viruses (e.g., HIV-1, Ebola virus, or Dengue virus) by blocking the interaction of Tsg101 protein, a member of the host endosomal sorting complex required for transport (ESCRT), with ubiquitin. Inhibition of Tsg101-ESCRT interaction blocks delivery of ESCRT to budding viral sites, causing the suppression of the virus particle release by membrane scission [45]. It is clear from these examples that different compounds, originally established as classical proton pump inhibitors, can interact with different cellular targets, resulting in a strong affecting on the viral replication process. This phenomenon probably also applies to diphyllin **1**.

Finally, we note that in our efforts to uncover the range and extent of the antiviral activities of diphyllin **1**, as described here, we also outlined a revised, updated, and improved synthesis of diphyllin **1** to support these in vitro studies. The further benefits of this synthesis are two-fold: (1) it will enable the cost-effective provision of much larger quantities of this compound for future in vivo follow-up experiments using animal models of virus infections; and (2) it will make possible the preparation of new in house diphyllin analogs suitable for future structure-activity correlation experiments. In particular, the synthesis will make possible the relatively facile preparation of new diphyllin analogs through functional group substitutions of at C-3, C-4, C-2′, C-3′, C-4′, C-5′, and C-6′ (see Figure 1A). We expect to report the results of such structure-activity correlation experiments in due course, building on the results of the studies described here.

## 5. Conclusions

We have prepared diphyllin **1** de novo, and this was shown to be a strong inhibitor of multiple viruses of the *Flaviviridae, Phenuiviridae, Rhabdoviridae,* and *Herpesviridae* families using Vero and BHK-21 viral infection cell models. These data convincingly suggest that diphyllin **1** is a broad-spectrum host-targeting antiviral agent blocking replication of multiple medically important enveloped RNA and DNA viruses. The next stage will be the implementation of in vivo model studies making use of drug delivery nanoparticles and other nanomedicine approaches to optimize antiviral effects while minimizing undesirable off-target cytotoxicities.

## Figures and Tables

**Table 1:** Cytotoxicities of diphyllin **1** and cleistanthin B **8** for multiple cell types after a 48 h incubation.

Cell Type	Diphyllin 1		Cleistanthin B 8	
	CC_50_ (µM) ^1,2^	95% CI (µM)	CC_50_ (µM) ^1,2^	95% CI (µM)
Vero (48 h)	>100	-	>100	-
Vero (72 h)	>100	-	>100	-
Vero (144 h)	18.19	15.1 to 21.65	80.73	66.54 to 104.2
PS (48 h)	92.44	62.9 to 140.5	78.86	48.17 to 129.40
UKF-NB-4 (48 h)	11.13	7.21 to 17.35	6.17	4.53 to 8.43
Huh-7 (48 h)	1.89	1.53 to 2.32	3.96	3.01 to 5.243
HBCA (48 h)	3.39	2.07 to 5.71	2.08	1.77 to 2.45
BHK-21 (72 h)	>100	-	-	-

^1^ Determined from three biological replicates of two independent experiments.^2^ Expressed as a 50% reduction in cell viability and calculated from the inflection points of sigmoidal percentage cell viability curves, which were obtained by a nonlinear fit of transformed inhibitor concentrations versus normalized response using GraphPad Prism 7.04 (GraphPad Software, Inc., USA).

**Table 2:** Antiviral and cytotoxicity properties of diphyllin **1** or cleistanthin B **8** in Vero (after a 48 h incubation) or BHK-21 cells (after a 72 h incubation).

Virus/Strain	Cell Type	Compound	EC_50_ (µM) ^1,2^	95% CI (µM)	CC_50_ (µM) ^1,2^	SI (CC_50_/EC_50_)
TBEV/Hypr	Vero	Diphyllin 1	0.85	0.47 to 1.42	>100	>117.64
TBEV/Hypr	Vero	Cleistanthin B 8	5.53	4.26 to 7.19	>100	>18.08
WNV/Eg-101	Vero	Diphyllin 1	0.78	0.52 to 1.15	>100	>128.20
ZIKV/Paraiba_01	Vero	Diphyllin 1	0.54	0.33 to 0.87	>100	>185.19
RVFV/H13/96	Vero	Diphyllin 1	0.46	0.29 to 0.70	>100	>217.39
RABV/CVS-11	BHK-21	Diphyllin 1	6.33	2.86 to 14.90	>100	>15.80
HSV-1/MacIntyre	Vero	Diphyllin 1	0.59	0.35 to 0.96	>100	>169.49

^1^ Determined from three biological replicates of two independent experiments. ^2^ Expressed as a 50% reduction in viurs titer or cell viability as calculated at inflection points of sigmoidal virus titer or percentage cell viability curves, which were obtained by a nonlinear fit of transformed inhibitor concentrations versus normalized response using GraphPad Prism 7.04 (GraphPad Software, Inc., USA).

## Data Availability

The datasets generated and analyzed during the current study are available from the corresponding author on reasonable request.

## Supplementary Materials

The following are available online at https://www.mdpi.com/article/10.3390/v14020354/s1, Figure S1: Functional comparison between our synthetic diphyllin **1** and the commercial product
