# Supplementary material for: Diphyllin Shows a Broad-Spectrum Antiviral Activity against Multiple Medically Important Enveloped RNA and DNA Viruses
Source: Viruses. 2022 Feb 9;14(2):354. doi: 10.3390/v14020354 (PMC8874615; doi:10.3390/v14020354)
Supplement: Supplementary file 1 [file viruses-14-00354-s001.zip › viruses-1552550-supplementary.pdf]

Supplementary information

Article

Diphyllin shows a broad-spectrum antiviral activity against multiple medically important enveloped RNA and DNA viruses

Michal Štefánik[1,2], Dattatry Shivajirao Bhosale[1,3], Jan Haviernik[1,4], Petra Straková[1,4], Martina Fojtíková[1], Lucie Dufková[1], Ivana Huvarová[1], Jiří Salát[1], Jan Bartáček[3], Jan Svoboda[3], Miloš Sedlák[3], Daniel Růžek[1,4,5], Andrew D. Miller[1,2,6*], and Luděk Eyer[1,4*]

1 Veterinary Research Institute, Emerging Viral Diseases, Hudcova 296/70, CZ-621 00 Brno, Czech Republic; stefanik@vri.cz (M.S.); bhosale@vri.cz (D.S.B.); haviernik@vri.cz (J.H.); strakova.petra@vri.cz (P.S.); fojtikova@vri.cz (M.F.); dufkova@vri.cz (L.D.); huvarova@vri.cz (I.H.); salat@vri.cz (J.S.); miller@vri.cz (A.D.M.); eyer@vri.cz (L.E.).

2 Department of Chemistry and Biochemistry, Mendel University in Brno, CZ-61300 Brno, Czech Republic

3 Institute of Organic Chemistry and Technology, Faculty of Chemical Technology, University of Pardubice, Studentská 573, CZ-532 10 Pardubice, Czech Republic; jan.bartacek@upce.cz (J.B.); jan.svoboda@upce.cz (J.Sv.); milos.sedlak@upce.cz (M.Se.).

4 Institute of Parasitology, Biology Centre of the Czech Academy of Sciences, Branišovská 1160/31, CZ-370 05 České Budějovice, Czech Republic; ruzekd@paru.cas.cz (D.R.).

5 Department of Experimental Biology, Faculty of Science, Masaryk University, CZ-62500 Brno, Czech Republic.

6 KP Therapeutics (Europe) s.r.o., CZ-61200 Brno, Czech Republic

* Correspondence: eyer@vri.cz (L.E.) and miller@vri.cz (A.D.M.).

A

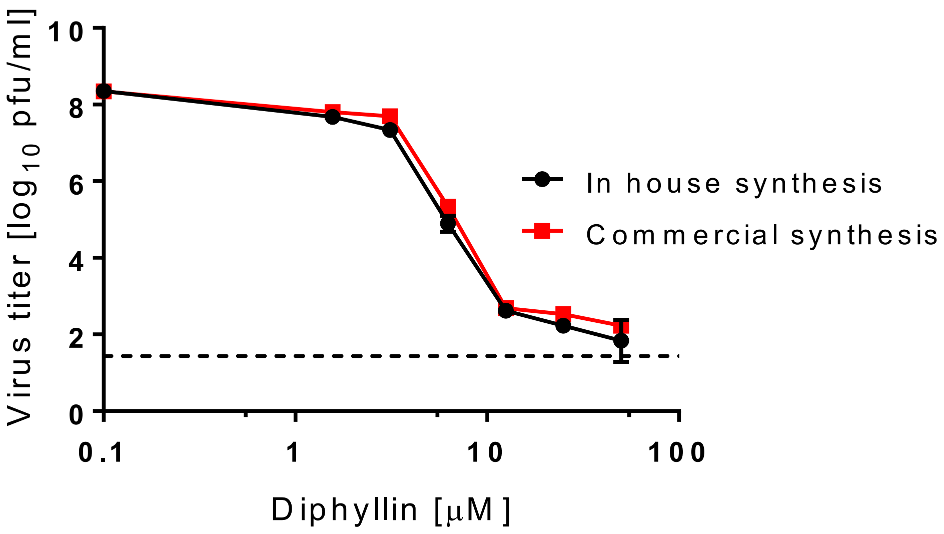

B

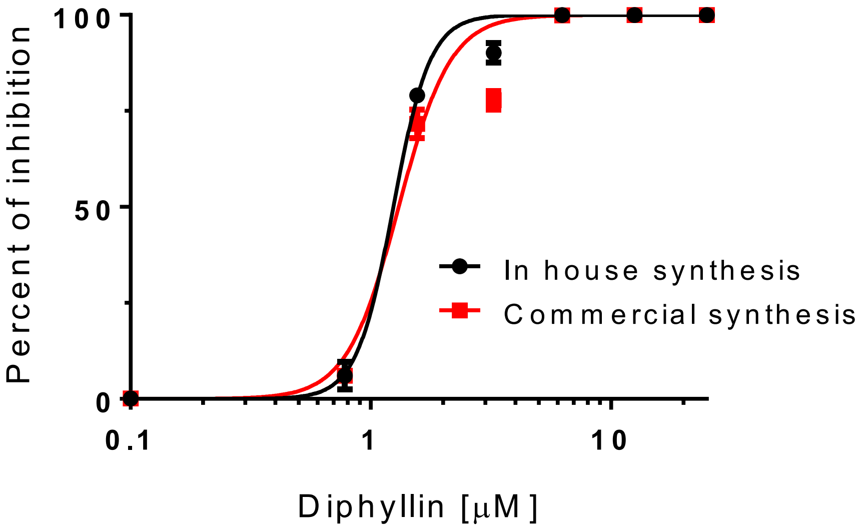

Figure S1. Functional comparison between our synthetic diphyllin **1** and the commercial product was carried out using an anti-TBEV antiviral assay based on PS cells. **(A)** Growth curves for TBEV (strain Hypr) cultivated in the presence of diphyllin **1**. Vero cell monolayers were treated with the in house synthesized diphyllin **1** or the commercially synthesized compound (0 to 50 μM) and infected simultaneously with TBEV (MOI of 0.1). The infected cells were cultivated for 48 h at 37 °C in 5 % CO_2, after which cell media were collected, and viral titers determined using a plaque assay and expressed as PFU/ml. **(B)** Inhibitory curves for the in house synthesized diphyllin **1** or the commercially synthesized compound (0 to 50 μM) constructed from the viral titers obtained in (A) and used for the calculation of EC_{50} values. The mean titers/percent inhibition from three biological replicates of two independent experiments are shown, and error bars indicate standard errors of the mean (n = 3). The horizontal dashed line indicates the minimum detectable threshold of 1.44 log_{10} PFU/ml.
